# Neurophysiological mechanisms of deep brain stimulation across spatiotemporal resolutions

**DOI:** 10.1093/brain/awad239

**Published:** 2023-07-14

**Authors:** Wolf-Julian Neumann, Leon A Steiner, Luka Milosevic

**Affiliations:** Movement Disorder and Neuromodulation Unit, Department of Neurology, Charité - Universitätsmedizin Berlin, Berlin 10117, Germany; Movement Disorder and Neuromodulation Unit, Department of Neurology, Charité - Universitätsmedizin Berlin, Berlin 10117, Germany; Department of Clinical and Computational Neuroscience, Krembil Brain Institute, University Health Network, Toronto M5T 1M8, Canada; Department of Clinical and Computational Neuroscience, Krembil Brain Institute, University Health Network, Toronto M5T 1M8, Canada; Institute of Biomedical Engineering, Institute of Medical Sciences, and CRANIA Neuromodulation Institute, University of Toronto, Toronto M5S 3G9, Canada

**Keywords:** deep brain stimulation, neuromodulation, mechanisms of action, singe neurons, oscillations

## Abstract

Deep brain stimulation is a neuromodulatory treatment for managing the symptoms of Parkinson’s disease and other neurological and psychiatric disorders. Electrodes are chronically implanted in disease-relevant brain regions and pulsatile electrical stimulation delivery is intended to restore neurocircuit function. However, the widespread interest in the application and expansion of this clinical therapy has preceded an overarching understanding of the neurocircuit alterations invoked by deep brain stimulation. Over the years, various forms of neurophysiological evidence have emerged which demonstrate changes to brain activity across spatiotemporal resolutions; from single neuron, to local field potential, to brain-wide cortical network effects. Though fruitful, such studies have often led to debate about a singular putative mechanism.

In this Update we aim to produce an integrative account of complementary instead of mutually exclusive neurophysiological effects to derive a generalizable concept of the mechanisms of deep brain stimulation. In particular, we offer a critical review of the most common historical competing theories, an updated discussion on recent literature from animal and human neurophysiological studies, and a synthesis of synaptic and network effects of deep brain stimulation across scales of observation, including micro-, meso- and macroscale circuit alterations.

## Teaser

Neumann *et al.* provide a critical review of competing theories of the mechanisms of action of deep brain stimulation. By integrating findings from neurophysiological studies across different spatiotemporal resolutions, they derive a potential explanation for how deep brain stimulation exerts its effects.

## Introduction

**Figure 1:**
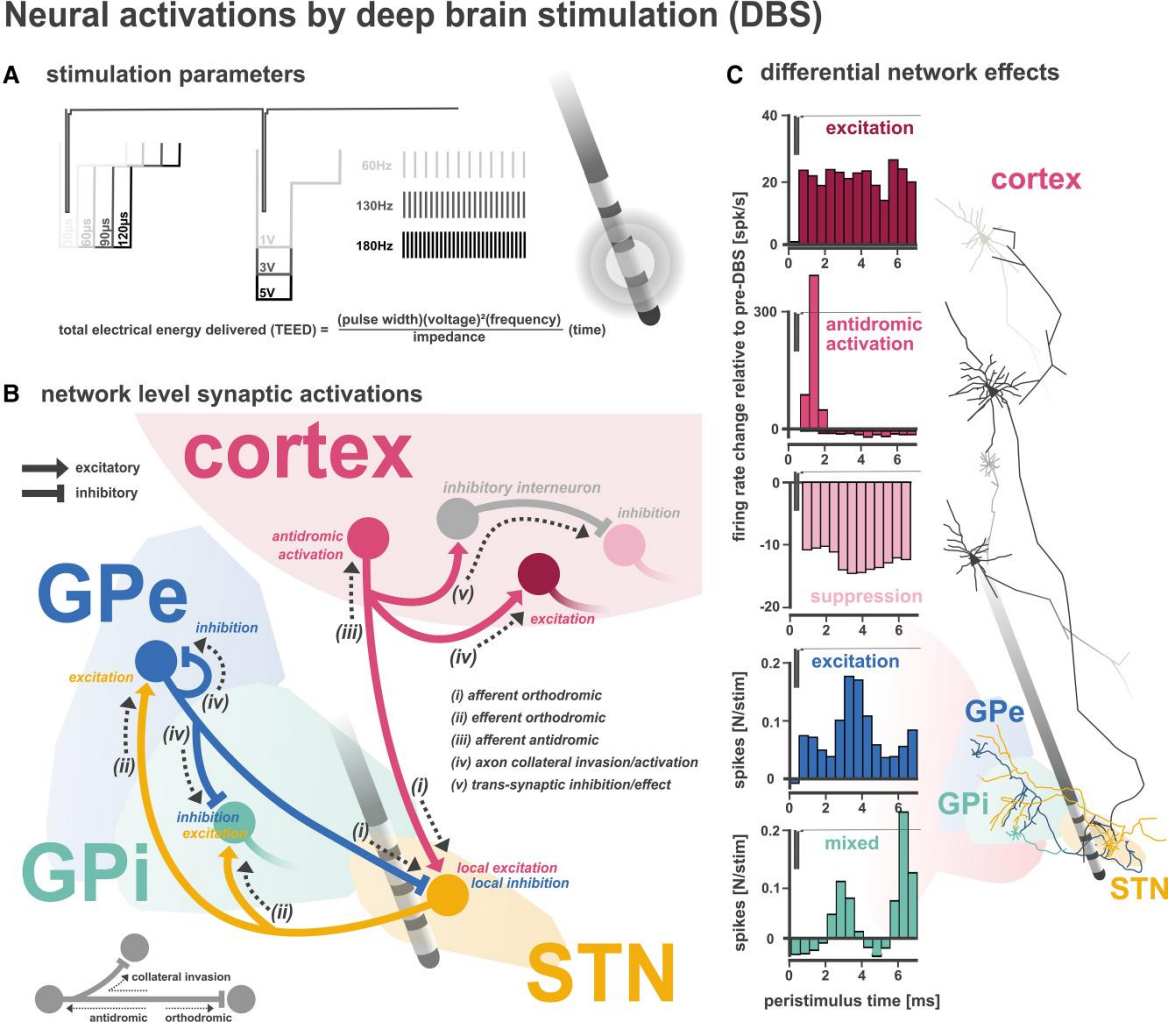
**Circuit engagements associated with subthalamic deep brain stimulation (STN-DBS).** DBS refers to pulsatile electric current applied through an implanted electrode.^1^ Typically, the contact expected to yield best clinical effects is set as the cathode, with the anode either set to the case of the implantable pulse generator (monopolar setting) or to another contact of the same electrode (bipolar stimulation).^2^ (**A**) In movement disorders, DBS is usually only effective when applied at high frequencies, with efficacy gradually increasing from 60 Hz up to or beyond 130–180 Hz.^3^ Typical pulse widths range from 30–200 µs. The pulse shape is rectangular and pseudo-monophasic with an initial square at therapeutic amplitudes of ∼1–5 V or 0.7–4 mA, followed by a passive charge balancing pulse with a several-fold longer pulse duration and lower amplitude. The total electrical energy delivered (TEED) is a function of the stimulation pulse width, amplitude, frequency and the impedance between the electrode contact and brain tissue.^4^ These parameters have significant influences on DBS effects^3^ by governing the extent of neural tissue engagement. (**B**) The stimulation activates neuronal structures, producing local and distant effects, which can include ‘orthodromic’ (downstream) and ‘antidromic’ (upstream) activation of afferent and efferent neuron projections, as well as ‘invasion’ of axonal architectures (i.e. activation of axonal branches/collaterals). The schematic takes into consideration common inputs and outputs of the STN and their collateral axonal branches to demonstrate the widespread effects of DBS. (**C**) Each plot shows the peristimulus spike firing histogram of engaged brain structures, demonstrating that STN-DBS can produce differential responses upstream in cortical regions (adapted from Johnson *et al*.^5^), as well as mixed responses in downstream structures such as globus pallidus internus (GPi) and externus (GPe) (adapted from Hashimoto *et al*.^6^). Single neuron traces of cortical^7^ (red), GPe^8^ (blue) and STN^8^ (yellow) are also depicted from three anatomical non-human primate tracing studies.

Deep brain stimulation (DBS) is a neurosurgical treatment that uses implanted electrodes and pulsatile electrical stimulation (Fig. 1A) to treat patients with Parkinson’s disease and other neurological and psychiatric disorders. Recent studies have provided significant insight into the neurophysiological effects of DBS, but the hope to find a single mechanism of action has often resulted in competitive and contradictory claims over the most relevant findings. No unifying theory on the causal therapeutic mechanism of DBS has won the consensus of experts, and it is now appreciated that the widespread effects are multifactorial.^1^ In the present Update, we aim to develop a holistic account on the current state of research on the neurophysiological mechanisms of DBS, with a focus on movement disorders. To dissect the effects of DBS, we must consider the affected circuits to which stimulation is applied. In Parkinson’s disease, loss of dopaminergic innervation leads to an imbalance of activity of the basal ganglia’s direct and indirect pathways (summarized in detail within Supplementary Fig. 1),^9^ producing over-inhibition of thalamocortical motor networks and a global network state change^10^ in the cortico-basal-ganglia-thalamo-cortical circuit. The primary target for DBS in Parkinson’s disease is the purportedly overactive subthalamic nucleus (STN), which exhibits widespread mono- and polysynaptic connections with distributed cortical and subcortical networks (Fig. 1B). In addition to the modulation of synaptic connections of the STN, DBS leads to parallel effects in multiple if not all nodes of these networks (Fig. 1C).^11^ We aim to provide an integrative account of complementary, instead of mutually exclusive, neurophysiological effects to derive a generalizable concept of DBS mechanisms. In particular, we offer a critical review of the most common competing theories of DBS mechanisms, an updated discussion on recent literature from human neurophysiological studies, and a synthesis of synaptic and network effects of DBS across scales of observation, including micro-, meso- and macro-scale circuit alterations.

## Common hypotheses on DBS mechanisms

### Local suppression

The first studies on the therapeutic effects of electrical stimulation with DBS in movement disorders were derived from a case series in patients who were undergoing stereotactic ablation of basal ganglia nuclei. Stereotactic radiofrequency ablation of the globus pallidus internus (GPi) and STN can alleviate motor signs of Parkinson’s disease^12^; a procedure currently being revived through the opportunity to perform lesions without opening the skull using magnetic resonance-guided focused ultrasound.^13,14^ The comparable clinical effects of DBS to pallidal and subthalamic lesions in human and animal studies led to the primary hypothesis that DBS causes reversible functional deafferentation of the neural target populations.^15^ An early study in patients with Parkinson’s disease showed that intraoperative microinjections of lidocaine (local anaesthetic) or muscimol (GABA agonist) to the STN led to a rapid reversal of parkinsonian symptoms in parallel with local suppression of neuronal firing in all three studied patients.^16^ These acute intraoperative effects resembled the subsequent therapeutic effects of high-frequency STN-DBS in these patients. Beyond the STN in Parkinson’s disease, suppression of local activity was also suggested as a primary mechanism of DBS in the pallidum and thalamus.^17–19^ The suppression of somatic firing had been hypothesized to be mediated by GABAergic activation,^20^ synaptic depression^21^ or depolarization blockade.^22^ The distant consequences of DBS in downstream subcortical nuclei were not easily accessible in patients, as neurophysiological recordings in structurally connected brain regions would require additional stereotactic trajectories beyond the therapeutic target. However, in addition to thorough characterizations of the cortical network effects of DBS in patients (summarized in the ‘Macroscale circuit effects’ section), a large body of computational modelling and preclinical animal work has focused on potential distant neural modulation by orthodromic and antidromic axonal activations.^23,24^ These works have often found more complex DBS effects than expected (Fig. 1C), which has cast doubt on the local suppression hypothesis.

### Information lesion

In non-human primates rendered parkinsonian by treatment with 1-methyl-4-phenyl-1,2,3,6-tetrahydropyridine (MPTP), short trains of subthalamic high-frequency stimulation (HFS) were reported to increase pallidal firing, which was incompatible with the hypothesis that STN-DBS suppresses all synaptic connections (in this case, glutamatergic excitation of the pallidum).^6^ Similarly, short durations of pallidal DBS lead to decreased firing of thalamic neurons,^25^ as opposed to the expected inactivation of GABAergic pallidothalamic projections. While DBS appeared to reduce somatic firing (local suppression hypothesis), these studies suggest that the efferent axons and their synaptic terminals could in fact be reliably activated by DBS pulses. This led to the decoupling of axon and soma hypothesis,^26^ and an adaptation of the local suppression hypothesis: the idea of an informational lesion^27^—an accepted yet relatively unspecific DBS mechanism, reminiscent of a new appreciation of the complexity of electrical stimulation effects on neural circuits. In recent neurophysiological works (detailed in the ‘Microscale circuit effects’ section), this complexity has been attributed to synapse-specific dynamics of short-term synaptic plasticity, in that afferent and efferent synapses can have unique functional properties (e.g. resilient versus vulnerable synaptic transmission fidelities). Recent studies have shown that local and antidromic firing can be increased in the short-term, but suppressed after longer stimulation periods,^5^ and it is therefore possible that high-frequency DBS cannot chronically sustain the high rate of activation associated with clinically effective stimulation over longer durations.^28^ This is further supported by *in vitro* studies that have reported that HFS of thalamocortical axons can lead to a rapid sigmoidal decrease of efferent layer 4 neuron firing through short-term synaptic transmission failure.^29^

### Antidromic activation

A series of rodent studies has suggested that antidromic activation of motor cortex may play a particularly important role in producing anti-parkinsonian effects in animal models.^30–33^ Low latency antidromic evoked potentials have also been reported in patients using invasive and non-invasive electrophysiology.^34–36^ However, the causal or mechanistic role of antidromic cortical activation in the clinical effect of DBS has been challenged by an important non-human primate report suggesting that antidromic activation is unstable over time, can shift to synaptic depression, and is only present during therapeutically effective STN but not GPi stimulation.^5^ Given that both STN and GPi DBS are effective for the alleviation of Parkinson’s disease motor signs, these findings are difficult to integrate with an important role of antidromic activation of cortex for the clinical treatment of Parkinson’s disease. Of note, short latency cortical evoked responses have been reported for GPi-DBS but were attributed to direct stimulation of the corticospinal tract instead of specific subcortical pathways, whereas long latency cortical evoked responses during GPi-DBS are thought to reflect feedforward/downstream basal-ganglia-thalamo-cortical network interactions rather than direct antidromic activation of cortico-basal ganglia fibres.^5,34^ Furthermore, one study showed that the effects of direct motor cortical stimulation have elicited improvements in the motor signs of patients with essential tremor, but not Parkinson’s disease,^37^ while other studies have shown only modest symptom alleviation in patients with Parkinson’s disease^38^ or transient beneficial effects in parkinsonian non-human primate models.^39^

### Action potential collision and axonal invasion

It must also be considered that axonal projections may be polysynaptic, with axonal branches terminating at different neural targets. Thus, in addition to ‘jamming’ incoming information through action potential collisions (via antidromic backpropagation of action potentials towards the soma),^24^ axonal activations may also ‘invade’ axonal branches of afferent and efferent projections, leading to neurotransmitter release at various terminal locations (summarized in Fig. 1B).^23^ For example, an STN-DBS pulse can produce an action potential upon a cortico-subthalamic fibre, which can antidromically backpropagate along the axon towards the soma of cortical neuron ‘A’; but, if the axon of ‘A’ also branches off to innervate cortical neuron ‘B’ (i.e. polysynaptic connection), then the A-B synapse can be activated via ‘invasion’ of the axonal collateral projection (see Fig. 1B inset for schematic).^23^ This invasion principle can also apply to subcortical circuitry leading to more complex effects, whereby STN-DBS pulses may simultaneously excite GPi or the substantia nigra pars reticulata (SNr; by way of STN efferent axonal activations) and inhibit GPi or SNr [by way of invading the axon collaterals of afferents from the globus pallidus externus (GPe)].^6,40^

### Mixed insights from optogenetics studies

Optogenetics is a powerful neuroscientific tool that allows for the manipulation of neuronal circuits *in vivo* with unprecedented, millisecond precision, enabling mechanistic studies of potential DBS effects in rodents.^41^ In 2009, Gradinaru *et al*.^30^ published a seminal report that optogenetic activation of cortico-subthalamic hyperdirect pathway axons in the acute 6-OHDA mouse model of Parkinson’s disease was necessary for therapeutic benefit, while direct inhibition of local STN neurons was not. However, stimulation of the hyperdirect pathway was not entirely dissociated from the inhibition/suppression of STN firing (as seen in their fifth figure). This may have been a result of activating both excitatory hyperdirect and inhibitory indirect pathway projections, or perhaps a result of inactivation/depression of excitatory cortico-subthalamic inputs. Nevertheless, more refined channel rhodopsins (i.e. optogenetics 3.0, referred to as the ‘dual virus approach’ that targets both projection source and target, with the opsin only expressed in neurons that get both) supported the therapeutic role of hyperdirect pathway axons in a mouse model of Parkinson’s disease.^31^ However, more elaborate behavioural analyses (spontaneous behavioural tests instead of amphetamine provoked) have also shown a direct beneficial effect of optogenetic inhibition or inactivation of the STN^42,43^ using the same opsin as Gradinaru *et al*.^30^ Similarly, next generation ultrafast opsins (i.e. Chronos) have provided evidence that optogenetic stimulation of STN neurons that resulted in bidirectional neuronal responses (53% of STN neurons showed increases in firing rate, while 32% showed decreases) could in fact also improve parkinsonian symptoms in rodents.^44^ Moreover, optogenetic interventions in GPe have produced long-lasting motor recovery in parkinsonian rodents, achieved by stimulating a subpopulation of parvalbumin-expressing GPe cells that preferably project to the STN.^45,46^ These studies have therefore provided evidence that activation of indirect pathway pallido-subthalamic projections may also hold therapeutic potential.

### Conclusion: complex and multifaceted

Ultimately, the mechanisms elicited by DBS have historically been considered as complex and varied; with effects that are parameter-dependent, site-specific, synapse-specific and likely more. Future studies investigating neural effects over longer time periods, with simultaneous recordings from multiple structurally and/or functionally connected regions, together with related clinical information, will have significant impact on our understanding on underlying brain network interactions.^47^ While optogenetics has provided a powerful means of circuit-level interrogation of the mechanisms of DBS, the results of such studies have similarly produced mixed insights, and it is moreover important to consider that these studies are putatively limited in translational value due to the level of invasiveness and manipulation required for further validation in humans. As such, the remainder of this article is focused on recent mechanistic insights gained from intraoperative and perioperative neurophysiological studies in DBS patients, highlighting the most recent mechanistic insights from the literature across various spatiotemporal resolutions.

## Microscale circuit effects: modulation of single neuron and synaptic activity

**Figure 2:**
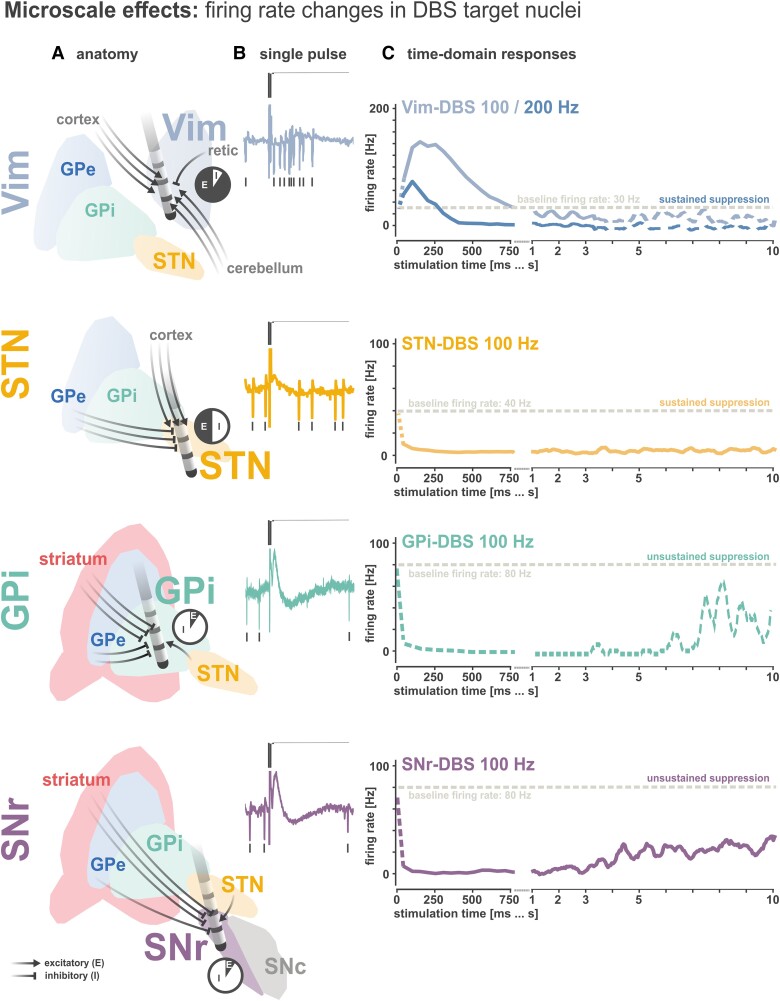
**Microscale effects of DBS.** The local neuronal effects of deep brain stimulation (DBS) have been proposed to be governed by activation of afferent inputs. (**A**) In the thalamic ventral intermediate nucleus (Vim) the vast majority of inputs are glutamatergic from the dentatorubrothalamic tract, with some GABAergic afferents from the thalamic reticular nucleus. In the subthalamic nucleus (STN), there are similar proportions of GABAergic (from globus pallidus externus, GPe) and glutamatergic (from cortex) inputs, somewhat favouring inhibitory inputs in number.^56^ In both globus pallidus internus (GPi) and substantia nigra parts reticulata (SNr), GABAergic projections (mainly from striatum and to a lesser degree from GPe) outweigh glutamatergic projections from STN. Pie charts depict estimated distributions of excitatory (E) versus inhibitory (I) afferent inputs. (**B**) The balance of GABAergic versus glutamatergic afferent fibres activated defines the net response to single pulses of stimulation, producing strong excitations in Vim, weak net inhibitory responses in STN, and strong inhibitions in GPi and SNr (adapted from Milosevic *et al*.^51,53,57^). (**C**) In Vim, high-frequency stimulation (HFS) produces an initial increase of firing, followed by a suppression of firing, which is stronger when higher stimulation frequencies are applied; hypothesized to be the result of synaptic depression (adapted from Milosevic *et al*.^55^; dashed time series is from *n* = 1 previously unpublished data demonstrating the persistence of neuronal suppression over time). HFS produces sustained inhibition of neuronal firing in STN due to persistent activation of GABAergic inputs, but incomplete neuronal suppression in SNr and GPi (*n* = 1; previously unpublished data depicted by dashed time series) where a return of neuronal firing can be observed over several seconds due to the depression of GABAergic inputs from striatum (STN and SNr figures adapted from Steiner *et al*.^58^). SNc = substantia nigra pars compacta.

Building upon previous modelling studies,^28,48–50^ an integrated computational framework^51^ has recently been developed that is capable of reproducing experimental results of stimulation induced local neuronal firing rate changes in human STN,^52,53^ SNr^53^ (or GPi^54^), and thalamic ventral intermediate nucleus (Vim)^55^ at various stimulation frequencies. The framework suggests that individual DBS pulses simultaneously activate the presynaptic terminals of all afferent axons terminating^49^ at the target structure. The consequent neuronal responses are thereby dependent upon the relative distributions of excitatory and inhibitory afferent inputs converging^50^ upon the target site (Fig. 2A). These neuronal responses to individual stimuli are furthermore embedded within a model of short-term synaptic plasticity,^48^ which is used to modulate the synaptic transmission fidelity of successive afferent input activations depending on the stimulation frequency. Therefore, this integrative model takes both neuroanatomical (local microcircuitry) and neurophysiological (short-term plasticity) properties into consideration to reproduce site-specific and frequency-dependent responses to DBS.

In targets with predominantly glutamatergic excitatory inputs (e.g. cortico- and rubro-thalamic projections to Vim), individual stimuli lead to a net postsynaptic depolarization and resultant stimulus-locked generation of action potentials.^51,55^ In stimulation targets with predominantly GABAergic inputs (e.g. striatal projections to GPi and SNr), individual DBS pulses lead to a net hyperpolarization and stimulus-locked inhibition of target neuron firing.^53,57^ STN has a more balanced distribution of glutamatergic (i.e. hyperdirect pathway) and GABAergic (i.e. indirect pathway) inputs, leading to weaker net inhibitory responses compared to SNr or GPi.^53,58^ These single-pulse phenomena (evoked fields and associated single neuron responses) are visualized in Fig. 2B. In the case of HFS, empirical data suggest that STN, SNr and GPi neurons become suppressed, while Vim neurons are initially excited, followed by a rapid attenuation of neuronal firing (Fig. 2C). In the model, this was accounted for by modulating the strength of consecutive postsynaptic activations in a frequency-dependent manner; which is the basis of short-term synaptic plasticity (i.e. transient/reversible changes to synaptic efficacy during repetitive stimuli). Namely, synaptic depression may explain the loss of synaptic efficacy during HFS, which can be caused by the depletion of presynaptic neurotransmitter vesicles, altered vesicle release dynamics, and/or reduction in presynaptic calcium.^59^

However, while the model could simulate the suppression of neuronal firing by non-selective synaptic depression of afferent currents (both excitatory and inhibitory), it has recently been suggested that the modulations of short-term synaptic dynamics differ across synapse types. In naïve rodent STN slices, DBS-like extracellular HFS rapidly and reliably depressed excitatory cortico-subthalamic synaptic currents; however, inhibitory GPe-STN synaptic transmission efficacy was maintained over time, after an initial weakening.^60^ This resiliency of inhibitory synaptic transmission to STN has now also been confirmed in patients with Parkinson’s disease.^58^ Importantly, this new empirical work emphasizes that differences in susceptibility to synaptic depression are not neurotransmitter-specific, but are in fact synapse-specific. While inhibitory synaptic transmission to STN (mediated by GPe) was partially sustained during subthalamic HFS, inhibitory synaptic transmission to SNr (mediated primarily by striatum) was rapidly depressed by SNr HFS.^58^ This potent depression of inhibitory synaptic fidelity during SNr HFS resulted in the return of spontaneous neuronal firing in SNr after several seconds, compared to sustained inhibition as seen in STN with STN HFS (Fig. 2C).

Ultimately, it can now be empirically supported that the effects of DBS are anatomy-dependent and synapse-specific; adding explicit mechanistic details to the otherwise unspecific claims of complexity. When taking each of these nuances into consideration (which synapses are engaged and how they behave), it is possible to conceive a generalizable theory as to how DBS may modulate neuronal activity. Mechanistic studies demonstrating the target- and circuit-specific effects of DBS^51,61^ furthermore emphasize that it may be possible to achieve more selective control of neural activity than anticipated with extracellular stimulation,^62,63^ particularly when strategically leveraging neurophysiological readouts to titrate stimulation location and parameter settings.^64^ Therefore, such functional readouts should be considered in the development of new and emerging DBS paradigms and indications.

Lastly, while the local neuronal suppression in STN and Vim has been suggested to be associated with clinical symptom alleviation in patients with movement disorders,^52,55^ these studies do not rule out the clinical importance of broader network-level effects. To this end, from a mechanistic standpoint, the aforementioned theoretical framework can be expanded upon to capture the simultaneously evoked (synapse-specific) up- and downstream responses at structurally connected targets (Fig. 1B and C). However, the inevitable causality of local versus distal effects on clinical benefit is an area of continued research interest. The following sections focus on mechanistic insights beyond the effects elicited upon stimulation target neurons.

## Mesoscale circuit effects: modulation of oscillatory local field potential activity

Beyond firing patterns, access to intracranial field potentials from DBS electrodes has defined research into disease and symptom specific pathological oscillatory patterns.^65^ The local field potential (LFP) represents a summation of presynaptic transmembrane voltage fluctuations, ion flux and local action potential firing.^66^ Thus, with LFP, synchronized neural population activity can be recorded that likely reflects pre- and postsynaptic currents from thousands of neurons surrounding the DBS electrode directly in the target region. Increased oscillatory power in the beta frequency band (13–35 Hz) was demonstrated to be related to the parkinsonian hypodopaminergic state,^67^ both in the STN and GPi,^68^ and the amplitude of these oscillations was shown to be correlated with parkinsonian motor sign severity in the dopaminergic OFF state.^69,70^

**Figure 3:**
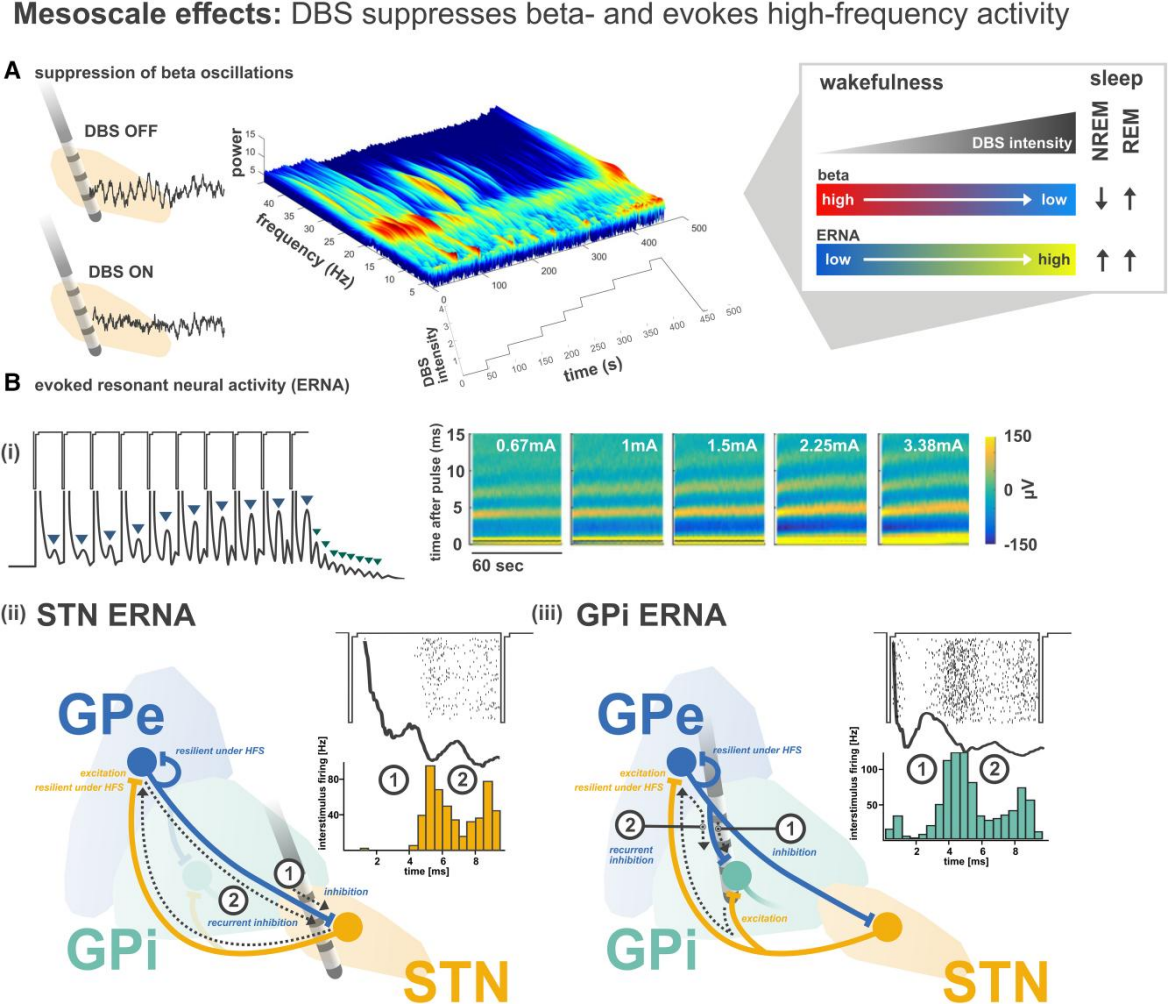
**Mesoscale effects of DBS.** Deep brain stimulation (DBS) can simultaneously suppress pathological oscillatory beta (13–35 Hz) activity in Parkinson’s disease and give rise to high-frequency evoked resonant neural activity (ERNA). (**A**) Time-frequency plot demonstrates changes to subthalamic beta oscillatory power with increasing DBS stimulation amplitude in an LFP recording from a parkinsonian patient OFF medication (adapted from Feldmann *et al*.^78^). (**B**) The ERNA waveform is observable (**i**) after each high-frequency pulse (blue downward arrows in waveform panel during stimulation; adapted from Sinclair *et al*.^92^) with decaying resonance even after stimulation is switched off (green downward arrows after stimulation). In tandem with the suppression of beta activity at higher stimulation intensities, the amplitude of ERNA increases (adapted from Sinclair *et al*.^90^). The schematics *below* demonstrate intraoperative ERNA waveforms and peristimulus spike firing histograms in both (**ii**) subthalamic nucleus (STN) and (**iii**) globus pallidus internus (GPi) during 100 Hz stimulation, as well as hypothesized circuit activation profiles that would explain the emergence of ERNA (adapted from Steiner *et al*.^93^). In STN, each stimulus would produce a net inhibitory response in STN, as well as concurrent excitation of globus pallidus externus (GPe), resulting in feedback inhibition in STN. The same is hypothesized for GPi ERNA via invasion/activation of collateral projections and axons of passage of the reciprocal STN-GPe connectivity. Thus, each of the ERNA waveform peaks is likely a substrate of inhibitory input via GPe. An important note is that the spike firing patterns in STN are only achieved when using subthreshold stimulation amplitudes, which do not cause complete suppression of neuronal firing. High-frequency stimulation (HFS) at clinically relevant intensities would result in the complete suppression of firing and the elicitation of large amplitude ERNA waveforms. In GPi, patterned firing seems to manifest when spike firing re-emerges (as shown in Fig. 2) after depression of striatal GABAergic inputs, likely unmasking inhibitory-excitatory GPe-STN competition, which could give rise to the mixed response as depicted in Fig. 1C. This phenomenon is currently under further investigation by the authors. The *inset* in the *top right* summarizes that stimulation intensity differentially modulates beta versus ERNA amplitudes, and furthermore emphasizes an additional potential use of ERNA as a closed-loop marker during NREM sleep, during which beta oscillations are attenuated.

Importantly, DBS was demonstrated to cause a local suppression of this pathological activity pattern^71–78^ (Fig. 3B), with the amount of beta suppression correlating with the magnitude of symptom alleviation.^79^ These observations have therefore given rise to the hypothesis that DBS acts through modulation of oscillatory patterns.^76,80^ While this has led to debate regarding the importance of single neuron ‘firing rate’ versus aggregate level LFP ‘oscillation’ changes^9,81,82^ as putative physiological signatures of Parkinson’s disease and DBS, cumulative evidence in fact supports a complementary and integrative nature of these two phenomena.^83^ In particular, ongoing LFP oscillations are encoded by phase-locked single-neuron burst firing patterns in the STN occurring at the same frequency,^84^ and similarly to LFP oscillations, pause burst firing patterns in the alpha and beta frequency bands correlate with Parkinson’s disease motor sign severity.^85^ Therefore, suppression of beta rhythmicity and exaggerated neuronal firing can go hand in hand, given that these phenomena are likely to be manifestations of one another at different spatiotemporal resolutions.^84^ While the exact relevance of oscillatory phenomena nevertheless remains disputed,^68,82^ the presence of beta activity in Parkinson’s disease and its suppression through DBS is a highly robust and reproducible finding. The most recent evidence suggests that prolonged episodes of elevated beta LFP power (> 900 ms) are particularly detrimental to the parkinsonian state.^70,86–88^ Conversely, antiparkinsonian medications and adaptive closed-loop DBS were retrospectively shown to shorten prolonged beta episodes, implying that short duration beta episodes may be of a more physiological nature.^86,89^

In addition to the suppression of pathological beta activity, a novel stimulation-evoked LFP marker of DBS response has recently been described, termed evoked resonant neural activity (ERNA).^90–92^ After each DBS pulse, a transient evoked phenomenon is visible, which typically contains two positive-going peaks in the 7 ms interstimulus window during 130 Hz stimulation, and remains observable for a short time period after cessation of stimulation [Fig. 3B(i)].^94^ ERNA has been postulated to be the result of entraining the inhibitory-excitatory reciprocal connections between GPe and STN.^95^ An ERNA substrate has now also been detected in the context of intraoperative microelectrode recordings, and it has been confirmed that each peak of the interstimulus waveform is associated with temporally-locked neuronal inhibition [Fig. 3B(ii)], with ERNA magnitude being positively correlated with the potency of inhibition.^93,96^ Mechanistically, it is hypothesized that the first ERNA peak is the result of a net inhibitory response from activating the afferent inputs to STN, while the resonant peak is the result of concurrent activation of STN efferents, which excite GPe, resulting in feedback inhibition to STN [Fig. 3B(ii)].^93,96^ While this theory is dependent upon the unique ability of GPe afferent synapses to maintain a high rate of activation,^58^ the same would need to be true for STN efferent synapses. To this end, it has indeed been shown that STN HFS results in persistent upregulation of GPe firing, albeit decaying over a slow time course.^6^ Connecting insights, it can now be confirmed that patterned interstimulus inhibitions of neuronal activity in STN (neuronal ERNA substrate in STN during HFS) [Fig. 3B(ii)] occur at almost exactly the same time as patterned interstimulus excitations of neuronal activity in GPe (depicted in Fig. 1C)^6^; further corroborating the reciprocal mesocircuit activation hypothesis. Moreover, through the use of a recently published expert anatomist-derived subcortical fibre atlas,^97^ it was shown that the ERNA hot spot in STN is in fact associated predominantly with the activation of reciprocal fibres between STN and GPe.^96^

Most recently, observations of ERNA have also been recorded by macrocontacts of the DBS electrode during GPi-DBS,^98^ and these observations have also been confirmed intraoperatively [Fig. 3B(iii)].^93^ Since various studies have shown that ERNA amplitude scales with therapeutic benefit of both STN-^64,92^ and GPi-DBS,^98^ ERNA may represent a convergent subcortical mechanistic signature, orchestrated by persistent GPe-mediated inhibition, that is able to explain the common efficacy of these two interventions. Given that hyperdirect pathway activation is not able to explain the common efficacy,^5^ ERNA may therefore impose a downstream influence on the greater basal-ganglia-thalamo-cortical network, which could explain the common cortical functional connectomic anti-correlation (a slower time course functional MRI-based readout), which co-localizes the predictive efficacy of STN- and GPi-DBS to the M1 region.^99^

Intriguingly, ERNA amplitudes have been found to correlate with amplitudes of beta oscillations,^90^ and the emergence of each of these signatures has been attributed to the same mesocircuit network.^95,96,100–102^ From an integrative standpoint, studies of these aggregate-level LFP markers therefore seem to imply that DBS replaces spontaneous pathological activity (i.e. increased beta oscillatory power) with evoked high-frequency activity by way of increased efficacy of GPe-mediated inhibition (which is otherwise pathologically downregulated in Parkinson’s disease). Both of these mesoscale signatures are presently under investigation in closed-loop DBS applications.^103,104^

## Macroscale circuit effects: modulation of distributed neurophysiological networks

**Figure 4:**
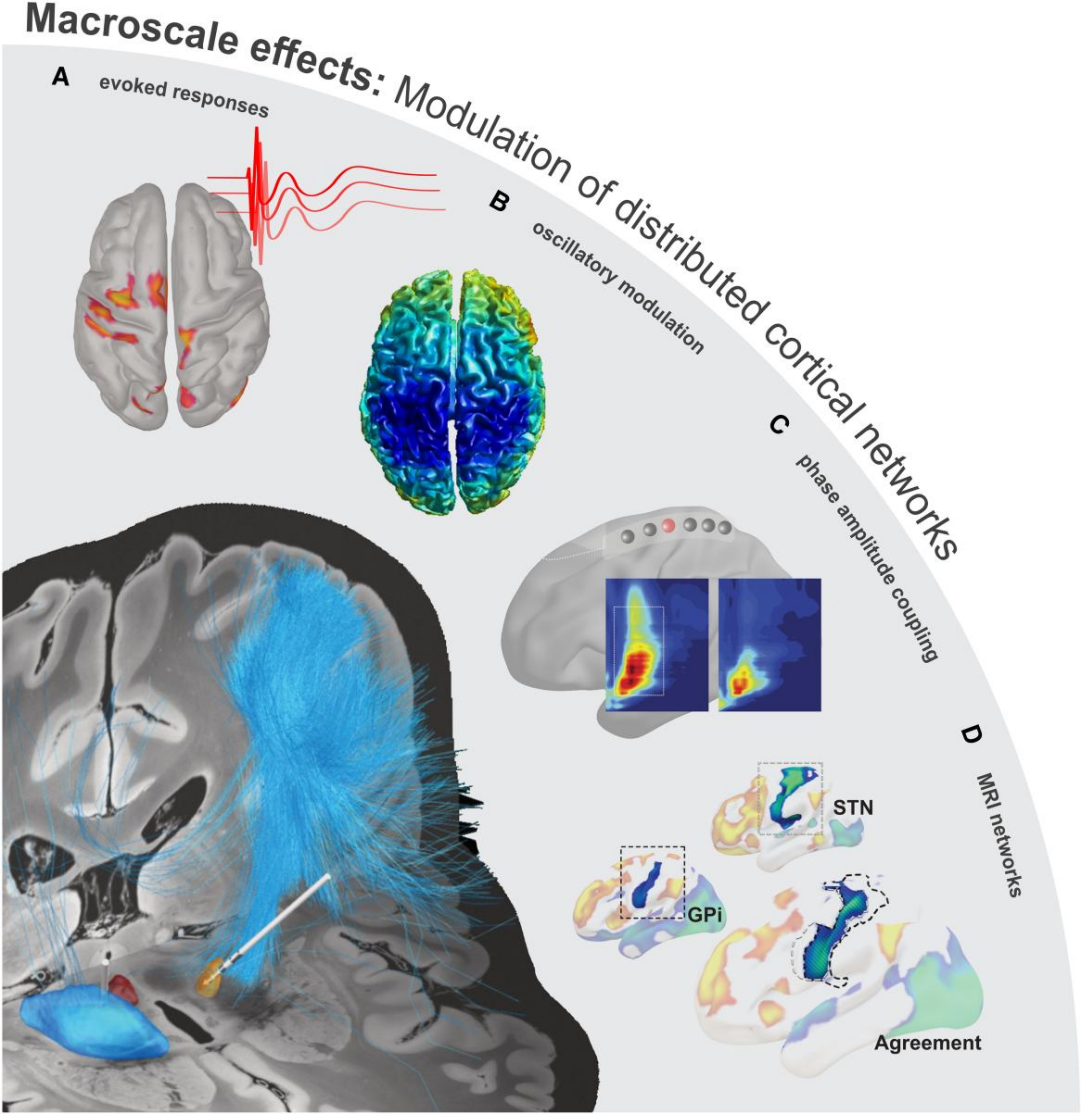
**Macroscale effects of DBS.** In addition to neurophysiological circuit alterations at the local level (i.e. subcortical functional changes at target structures), deep brain stimulation (DBS) has an influence upon whole-brain interregional networks. The modulation of a single node of a circuit will likely lead to state changes in all circuit connections. This could, in part, be achieved by local axonal activations (*bottom left*), as evidenced by (**A**) short-latency stimulation-induced evoked potentials in the cortex^128,129^; wherein the strength of network engagement (evoked potential amplitude) may be reflective of the achieved anti-parkinsonian efficacy with therapeutic settings (adapted from Bahners *et al*.^128^). Moreover, in addition to the suppression of subcortical beta oscillations, (**B**) DBS can suppress beta power across the sensorimotor cortical network (adapted from Abbasi *et al*.^109^), as well as (**C**) attenuate cortico-cortical beta-gamma coupling (adapted from De Hemptinne *et al*.^112^), which is otherwise pathologically elevated in Parkinson’s disease.^110^ Despite such findings, direct antidromic activation of cortical circuitry is suggested not be able to corroborate the clinical efficacy of GPi-DBS.^5^ (**D**) As such, the spatial overlap in functional connectivity, which co-localizes the predictive efficacy of STN- and GPi-DBS to the M1 region may be representative of a slower timescale (functional MRI-based) functional readout of a downstream basal-ganglia-thalamo-cortical network effect, which could be generated by a convergent GPe-mediated subcortical network activation profile achieved by STN- and GPi-DBS (i.e. ERNA). GPe = globus pallidus externus; GPi = globus pallidus internus; STN = subthalamic nucleus.

In addition to intracranial recordings from subcortical structures, it is possible to record broader network level cortical activity during DBS (Fig. 4).^105,106^ To this end, it was shown that the suppression of beta activity can be observed not only in target nuclei (STN or GPi), but also in distant cortical sensorimotor networks.^107^ Several studies using EEG, magnetoencephalography (MEG) and electrocorticography (ECoG) have independently replicated findings that cortical beta is modulated by STN-DBS in Parkinson’s disease.^79,108,109^ Beyond beta suppression, it was further shown that more complex neurophysiological phenomena can be observed in response to DBS. Namely, ECoG and EEG studies have shown that cortical beta-gamma cross-frequency coupling was increased in Parkinson’s disease^110,111^ and suppressed with DBS.^112^ It was moreover reported that cortical broadband activity was coupled to specific phases of the beta oscillation.^110,112^ Later, some of these observations were explained by a change of waveform shape of beta itself. Cycle-by-cycle waveform investigations^113,114^ have revealed that particularly sharp signal deflections are shorter and therefore less likely to nest broadband signals during DBS. Thus, increased sharpness asymmetry of trough and peak of the beta oscillation in Parkinson’s disease was shown to be smoothed during therapeutic DBS.^115,116^ In fact, specifically sharp single cycle discharges in the beta oscillation could be isolated in the parkinsonian motor cortex and associated with the hypodopaminergic OFF state.^116,117^ More recently, altered cross-frequency coupling in patients with Parkinson’s disease undergoing STN-DBS was also described using ECoG and EEG.^118^ Namely, coupling of finely tuned cortical gamma to the stimulation frequency and lower harmonics can be observed during DBS and may reveal underlying mechanisms of symptom alleviation.^118,119^ These effects have been hypothesized to be the result of a dynamic entrainment mechanism of neural populations to the stimulation frequency described in regions of a cortical (motor cortex, supplementary motor area, premotor cortex) and subcortical (STN and cerebellum) network. In particular, implementation of a Wilson-Cowan model (commonly used mean field computational model with local reciprocal excitatory-inhibitory network connectivity) was able to be replicate these gamma entrainment responses^120^; however, empirical neuronal/synaptic underpinnings are yet to be established, though these observations are perhaps reminiscent of the mechanistic underpinnings of ERNA (described earlier).

Network-level studies demonstrate that modulation of one node in the circuit can lead to distinct neurophysiological alterations recorded in other nodes of the same network. But beyond local subcortical or cortical changes in oscillatory activity, neurophysiology also allows for the investigation of functional oscillatory connectivity.^121–123^ The most comprehensive account of this was reported in a study in subthalamic DBS for Parkinson’s disease using combined MEG-LFP recordings.^79^ During DBS, it was shown that beta-band coherence (a measure of frequency specific correlation of phase and power between two recordings) can be suppressed by DBS, an effect that localizes to the medial frontal cortex overlapping with the supplementary motor area (SMA).^79^ In addition to synchrony, oscillatory networks can also be investigated for directionality of information flow, e.g. using Granger causality-based metrics. In patients with Parkinson’s disease, subthalamic beta activity is hypothesized to be driven by cortex. The most recent multi-modal work^124^ (combining MEG, LFP and computational modelling) suggests that cortical high-frequency beta oscillations (20–35 Hz) are propagated to STN, where they are subsequently converted to the more pathological low-frequency (13–20 Hz) rhythm^125,126^ by the reciprocal connectivity of the STN-GPe network; a phenomenon that may be exacerbated in the hypodopaminergic state.^110,123^ Importantly, while DBS modulates synchrony between cortex and STN, it does not change directionality of this network that follows the neuroanatomical/axonal orientation of the hyperdirect pathway.^79,124^

Finally, through recent functional connectomic works (investigation of functional MRI-based signatures of circuit activation), widespread changes to brain circuitry have been shown to converge on a common network that can be remarkably predictive of therapeutic success; with a particular emphasis on the functional anticorrelation between subcortical targets (STN and GPi) and primary motor cortex.^99,127^ Achievement of this optimal network profile can in fact result in the restoration of neurocircuit function that closely resembles that of age-matched healthy controls,^11^ which represents the ultimate goal of DBS as a neuromodulatory intervention.

## Conclusion

The effects of DBS are dependent upon the therapeutic target and the specific synapses that are present within the vicinity of the electrical field that are engaged by the stimulation pulses. While the distribution of GABAergic versus glutamatergic afferent inputs may determine the net neuronal response to individual pulses (i.e. selective excitation versus inhibition),^51^ the unique functional properties of engaged synapses seem to govern the response at high frequencies of stimulation^58^; underscoring the importance of understanding the short-term synaptic dynamics of engaged synapses on a structure-by-structure basis. In the case of STN-DBS, HFS seems to depress/abolish the cortical excitatory influence on STN, while promoting/persistently eliciting GPe-mediated inhibition.^58,60^ The suppression of cortical inputs could explain aggregate-level observations of the attenuation of pathological beta frequency oscillations, which are hypothesized to be dependent on cortical influence of the STN via the hyperdirect pathway projections.^124^ Conversely, the persistent supraharmonic entrainment of GPe-mediated inhibition (i.e. ERNA) may prove to be an important therapeutic mechanism, which seems to be able to explain the common clinical efficacy of STN- and GPi-DBS^64,93,98^; which cannot otherwise be explained by antidromic activation of the hyperdirect pathway.^5^ To this end, it is perhaps subcortical alterations that give rise to broader, downstream basal-ganglia-thalamo-cortical network level changes, which functionally co-localize the predictive efficacy of STN- and GPi-DBS to the M1 region (a functional MRI-based slow timescale functional readout).^99^ Meanwhile, invasion of the cortico-subthalamic hyperdirect pathway may explain faster timescale functional readouts such as the disruption of excessive oscillatory power^124^ and cortico-cortical coupling^79,112^ achieved by STN-DBS in Parkinson’s disease.

## Supplementary Material

awad239_Supplementary_Data
