# Supplementary material for: Neurophysiological mechanisms of deep brain stimulation across spatiotemporal resolutions
Source: Brain. 2023 Jul 14;146(11):4456–68. doi: 10.1093/brain/awad239 (PMC10629774; doi:10.1093/brain/awad239)
Supplement: awad239_Supplementary_Data [file awad239_supplementary_data.pdf]

Supplementary Material

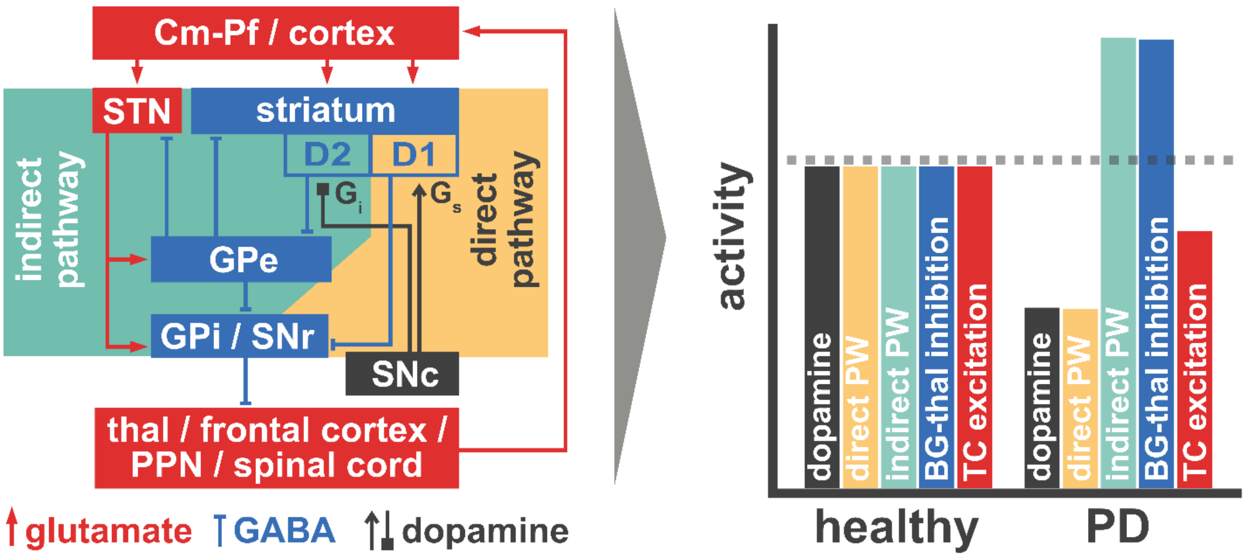

Supplementary Figure 1 – Basal ganglia (BG) circuit model in health and Parkinson's disease (PD). In PD, degeneration of dopaminergic substantia nigra pars reticulata (SNc) neurons results in the following phenomena. (i) Decreased inhibition of D1-mediated striatal projections to the substantia nigra pars reticulata (SNr) and globus pallidus internus (GPi); via the "direct pathway." (ii) Increased activity of D2-mediated striatal projections to the globus pallidus externus (GPe), which disinhibits the subthalamic nucleus (STN), causing overexcitation of GPi and SNr; via the "indirect pathway." Cumulatively, as a result of (i) *and* (ii), the BG output structures (GPi and SNr) over-inhibit thalamocortical (TC) motor networks, which is thought to give rise to the hypokinetic symptoms of PD.
